# Genetic Mechanisms and Aberrant Gene Expression during the Development of Gastric Intestinal Metaplasia and Adenocarcinoma

**DOI:** 10.2174/138920207783406460

**Published:** 2007-09

**Authors:** K Holmes, B Egan, N Swan, C O’Morain

**Affiliations:** Department of Clinical Medicine, Trinity College Dublin, The Adelaide and Meath Hospital, Tallaght, Dublin 24, Ireland

**Keywords:** Intestinal metaplasia, gastric cancer, aberrant gene expression, genetic markers.

## Abstract

Gastric adenocarcinoma occurs *via *a sequence of molecular events known as the Correa’s Cascade which often progresses over many years. Gastritis, typically caused by infection with the bacterium *H. pylori*, is the first step of the cascade that results in gastric cancer; however, not all cases of gastritis progress along this carcinogenic route. Despite recent antibiotic intervention of *H. pylori* infections, gastric adenocarcinoma remains the second most common cause of cancer deaths worldwide. Intestinal metaplasia is the next step along the carcinogenic sequence after gastritis and is considered to be a precursor lesion for gastric cancer; however, not all patients with intestinal metaplasia develop adenocarcinoma and little is known about the molecular and genetic events that trigger the progression of intestinal metaplasia into adenocarcinoma. This review aims to highlight the progress to date in the genetic events involved in intestinal-type gastric adenocarcinoma and its precursor lesion, intestinal metaplasia. The use of technologies such as whole genome microarray analysis, immunohistochemical analysis and DNA methylation analysis has allowed an insight into some of the events which occur in intestinal metaplasia and may be involved in carcinogenesis. There is still much that is yet to be discovered surrounding the development of this lesion and how, in many cases, it develops into a state of malignancy.

## 1 INTRODUCTION

### 1.1 Intestinal Metaplasia

Intestinal metaplasia (IM) is the term used to describe premalignant lesions of the stomach that are often present before and during the development of gastric cancer (GC). Precancerous or premalignant lesions are defined as lesions that precede invasive cancers in which many of the genetic abnormalities and phenotypic characteristics of invasive cancer are present but not yet fully expressed [1]. In 1955, Morson first described the progression of gastric intestinal metaplasia into gastric epithelial dysplasia and the subsequent development of gastric adenocarcinoma [2, 3]. Gastric tissues exposed to mutagens and/or carcinogens resulted in development of IM alongside GC; IM development was also demonstrated in rats following administration of N-methyl-N’-nitro-N-nitroguanidine *via *drinking water [4, 5], and following exposure to radiation [6]. In contrast, it has been proposed that IM may not always be a premalignant condition in GC development, but rather that intestinal-type cells in IM and GC may occur independently of one another [7].

### 1.2 Gastric Cancer and the Correa’s Cascade of Carcinogenesis

Gastric adenocarcinoma remains the second leading cause of cancer death worldwide, accounting for approximately 10% of all newly diagnosed cancers [3]. It is the most common form of malignant gastric tumor, accounting for about 90% of stomach cancers. Lack of early diagnosis is responsible for the high mortality rates as the disease is usually incurable when diagnosed at an advanced stage with a 5-year survival rate ranging from 5-15% [8]. Clinical outcome is therefore optimized by early stage diagnosis [1]. There is a recognized need to be able to identify and eliminate precursor lesions in order to treat many cancers, and a significant reduction in the mortality rate of GC could be achieved by the implementation of early diagnostic methods. There are two main types of gastric adenocarcinoma according to the Lauren classification defined as intestinal-type and diffuse-type. These histopathological variants have been shown to differ greatly in the phenotypic characteristics at the precursor stage [9]. Intestinal-type carcinomas display obvious glandular differentiation and arise from gastric cells that have undergone IM through a series of histological changes known as the Correa’s Cascade of Gastric Carcinogenesis. Diffuse-type carcinomas are typically poorly differentiated and may arise from either native gastric cells or those that have undergone IM [10], there are no known defined precursor lesions for this type of cancer [11, 12]. The Lauren classification is widely used as it describes two biological entities that are different in epidemiology, etiology, pathogenesis and behaviour. The Correa’s Cascade of Gastric Carcinogenesis is a model that refers only to intestinal-type carcinoma. The cascade outlines a process of carcinogenesis which is a gradual transition from initial gastritis to diffuse, chronic gastritis, mucosal atrophy, intestinal metaplasia, dysplasia, and finally, carcinoma [1, 13, 14]. The initiation of gastritis in the stomach is often caused by infection with *Helicobacter pylori*, a bacterial pathogen that frequently colonizes the intestinal mucosa of humans and animals. The organism may be carried asymptomatically for many years; however, it is also a recognized cause of chronic gastritis, gastric ulcers and gastric adenocarcinoma. The evidence linking this pathogen to GC led to *H. pylori* becoming classified as a class 1 carcinogen by the World Health Organization [15]. Fig. (**1**) shows a diagrammatic representation of the carcinogenic cascade, this diagram is a modification of the “hypothesis of gastric cancer etiology” as proposed by Correa [16]. Fig. (**2**) shows the pathology of the key steps along the Correa’s cascade of carcinogenesis. H&E stained paraffin-embedded tissue sections are presented, starting with normal gastric mucosa, Fig. (**2a**), followed by initial *H. pylori*-associated gastritis in Fig. (**2b**) and progression into IM in Fig. (**2c**), and finally intestinal-type GC in Fig. (**2d**).

Human stomach carcinogenesis is a multistep process involving genetic instability and numerous genetic and epigenetic changes in oncogenes, tumor-suppressor genes, cell-cycle regulators, cell adhesion molecules and DNA repair genes. The two different histological types of cancer: gastric and diffuse, arise from different combinations of these changes and steps; Tahara *et al. *[17] were amongst the first to determine the pathway of genetic events for the two types of cancer [12]. Gastric adenocarcinoma may further be categorised as “mixed” type consisting histologically of a combination of intestinal and diffuse type. The variation of complex phenotypes both during and preceding the development of GC has led to substantial research interest in gene expression. There have been many recent studies on the regulation of genes and their expression in many types of cancer, including GC; however, there are limited studies to date on the expression and regulation of genes during the precursor stages of GC. Recent research progress on gene expression and regulation in GC will be discussed in section 5.

## 2 MOLECULAR MARKERS OF IM TYPE AND CARCINOGENIC PROGRESSION

### 2.1 IM Classification

Further to the Lauren classification of types of gastroadenocarcinoma which are described as intestinal-type and diffuse-type, IM has more complex classifications which begin initially with two types: small-intestine-type and colonic-type. IM develops when gastric stem cells are diverted from the production of normal gastric cells (eg. surface cells, chief cells, parietal cells) towards the production of cells typical of the small or large intestine (eg. absorptive cells, goblet cells, paneth cells) [18]. Classification of IM into complete and incomplete types depending on the presence of paneth cells was first proposed by Kawachi and colleagues [19], and a further classification based on mucin secretion patterns and morphology defined small intestine-type and colonic-type. Three classes of IM were described by Jass and Filipe [20] based on morphology and classical mucin-staining using periodic acid-Schiff, Alcian blue and high iron diamine staining methods: Type I, Type II and Type III. Type I is described morphologically as complete IM containing all intestinal cell types including absorptive cells, with predominantly sialomucin- and some sulphomucin-secreting goblet cells. Type II is described as incomplete IM that lacks recognizable absorptive cells, and has only sialomucin-secreting goblet cells. Types I and II are also described as small-intestine-type. Type III is described as incomplete with only sulphomucin-secreting goblet cells and is also the colonic-type rather than small-intestine type. Type III IM confers a 4-fold greater risk of developing GC than Type I [18,  20].

These IM classifications are generally accepted yet they are only based on intestinal, and not gastric, properties of the cells [19, 21]. This has led to more recent improved classifications of IM consisting of gastric or G-type, intestinal or I-type and mixed gastric-intestinal or GI-type, based on the cellular origin of the cancer cell [19, 21]. Recent studies used immunohistochemical analyses to define the precursor lesion type; the type of IM can have significant implications in predicting progression of disease, for example, Type III IM confers a 4-fold greater risk of GC than does Type I IM.

Immunohistochemical analysis of the two phenotypically distinct types of cancer precursor lesions has led to the identification of a number of histological “markers” that can be used to determine whether GC, and indeed IM itself at the early stages, is of the gastric or intestinal type [3, 7, 21, 23, 24]. Progress with this has accelerated with microarray technology to determine a small profile of gene expression changes that correlates with progression of IM into gastric adenocarcinoma [8].

### 2.2 Genetic Markers of IM

Gene markers that have been identified as indicators of the progression and type of IM are cdx1 and cdx2, sox2, muc 2, muc5AC, muc6, claudin-4, mkk4, stratifin, villin-1, and hif-1α [3, 11, 19, 21, 23-31]. The caudal related homologue genes (cdx1 and cdx2) are evolutionarily conserved both in molecular structure and function and have critical function during the development of both foetal and adult intestinal epithelia. When expressed, they modulate proliferation, apoptosis, cell-adhesion, and columnar morphology. They are also necessary for expression of intestine-specific genes and promotion of the mature intestinal phenotype. Although several studies suggest that cdx homologues may act as tumor suppressors in the colon, ectopic expression of cdx1 and cdx2 in the gastric mucosa is involved in the development of precancerous IM [30].

Silberg *et al. *[25] first demonstrated that ectopic expression of cdx2 directed to the gastric mucosa of transgenic mice induced gastric IM. Cdx2 expression in gastric mucosa was achieved through the derivation of transgenic mice in which the mouse cdx2 cDNA was under the control of cis-regulatory elements of the transcription factor foxA3 in a yeast artificial chromosome (YAC). Histological examination of the gastric mucosa of the foxA3/cdx2 mice revealed the presence of alcian blue-positive intestinal-type goblet cells along with the RNA expression of the intestine-specific genes villin, fabpi, tff3, gcc and muc2, as detected by Northern blot analysis. Tff3 and muc2 are normally expressed in goblet cells in the small intestine and colon; fabpi is normally expressed in both absorptive enterocytes and goblet cells; villin is expressed in microvilli, which are present predominantly on the apical surface of goblet cells and absorptive enterocytes. The mice developed an IM phenotype with predominantly sulphated mucins; this is consistent with a Type III human gastric IM, where sulphated mucins are the predominant type [25]. The histological and molecular changes induced by cdx2 expression in mice mimic the changes that take place during the development of human IM, that is, the appearance of goblet cells and the activation of intestinal genes in the gastric epithelium. This study demonstrated the first causal link between cdx2 expression and IM [25].

Many subsequent studies have demonstrated the role of the cdx1 and cdx2 transcription factors in the initiation of IM. Their role as transcriptional activators of other intestine-specific genes has also been widely reported. Almeida *et al*. [23] performed immunohistochemistry staining of tissues from a cohort of patients with IM, gastric adenocarcinoma and a range of asymptomatic and dyspeptic patients. Staining was used to detect Cdx1, Cdx2, Muc2 and Muc5AC and it was revealed that Cdx1 and Cdx2 were consistently expressed in IM and in a subset of gastric carcinomas. They reported that Cdx1 and Cdx2 expression was independent of the type of IM and of gastric carcinoma, and that it was significantly associated with expression of the intestinal mucin Muc2 [23]. Further to this, Mesquita *et al. *[27] evaluated the role of cdx-1 and cdx-2 in muc2 transcription using co-transfection experiments with expression vectors encoding Cdx-1 and Cdx-2. Following successful transactivation of the muc2 promoter with expression vectors, gel-shift assays were used to identify two Cdx2 binding sites at –177/-171 and –191/-187. Mutation of these two binding sites subsequently abolished transactivation by Cdx-2, thus demonstrating a functional role for Cdx2 in regulating Muc2 mucin gene promoter activity [27]. A further study demonstrates that the cdx2 transcription factor regulates furin expression during intestinal epithelial cell differentiation; furin activity is essential for morphological differentiation of intestinal epithelial cells [31].

### 2.3 Improved IM Classification and Identification of Further Gene Markers

To further clarify the molecular mechanisms underlying the development of IM, a new classification was proposed to first provide a better explanation of the phenotypic status [19, 21, 24]. This takes into account both the gastric and intestinal properties of IM, thus dividing IM type into two major groups; a gastric/intestinal (GI)-mixed type, and a solely-intestinal (I) type. The morphological and biochemical differentiation status toward “gastric” and/or “intestinal” provide fundamental information for this classification system making gastrointestinal markers important to judge the progression. The first morphological change is the appearance of intestinal goblet cells and changes occur in mucin-type molecules. The muc5AC and muc6 genes of the gastrointestinal tract are mainly expressed in gastric foveolar mucosa and pyloric glands, respectively. The muc2 gene encodes a typical secretory gel-forming mucin, which represents the predominant form in human intestinal tissues. These genes and their consequent morphological changes are useful markers to distinguish between a gastric and intestinal phenotype. Sox2 is proposed as a potential marker for the gastric phenotype as transcript and protein expression was demonstrated in human stomach both with and without IM [21, 24]. A transcriptional expression profile was characterized for normal gastrointestinal tract isolated stomach glands from patients with a range of IM types including G, GI-mixed and I types. RT-PCR was used to detect expression levels of muc5AC, muc6, muc2, villin-1, cdx1, cdx2 and sox2. In normal gastrointestinal tract, the expression of muc5AC and muc6 was higher in the stomach than in intestine and conversely, muc2 and villin-1 expression levels were greater in the intestine than in the stomach. In G type glands, muc5AC and muc6 showed the greatest levels of expression and those of muc2 and villin-1 were significantly lower. Expression of muc5AC and muc6 gradually decreased during the intestinal shift from G to GI, and from GI-mixed to I type during IM progression. Simultaneously, muc2 was upregulated during the entire intestinal cell shift, but villin-1 was only upregulated from G to I, and not from GI to I type. Sox2 expression also gradually decreased as cdx 1 and 2 expression increased during the transition of IM from G to GI and to the I type. This study defines a set of gastrointestinal differentiation marker genes that can be used to assess the type and direction of progress of IM in immunohistochemical and molecular biological studies [21, 24].

The mucins are well recognized as important marker molecules for the classification and progression of IM of the stomach [32, 33], as well as having defined expression patterns according to the presence or absence of *Helicobacter pylori* infection [34, 35]. Mucin immunohistochemistry might feasibly replace classic histochemistry for the classification of IM into complete and incomplete types [32]. Many studies correlate the definitions of these IM types according to mucin expression: in type II/III or incomplete IM the mucins usually expressed in the stomach (muc1, muc5AC and muc6) are present; in type I or complete IM, muc2 is aberrantly expressed and there is decreased or absent expression of muc1, muc5AC and muc6 [21, 24, 32-34, 36]. The presence or absence of *H. pylori* infection has also been shown to correlate with mucin expression and onset of IM; IM usually leads to clearing of *H. pylori*. Where IM and *H. pylori* are found together, a particular pattern of incomplete IM is apparent, with expression of muc1 and muc5AC and with little or no expression of muc2. Where IM is present but *H. pylori* is absent, high levels of muc2 expression has been demonstrated regardless of the IM type [34]. A study on the different mucins expressed in different types and stages of adenocarcinoma suggested that the cellular mucin phenotypes result from distinct genetic alterations. Tumors were subclassified into 4 groups: gastric-phenotype, intestinal-phenotype, mixed-phenotype and unclassified-phenotype. Immunohistochemistry was used to detect chromosomal allelic losses of cancer-related loci, microsatellite instability (MSI), and overexpression of the p53 protein. The frequency of 3p allelic loss was higher than other markers in gastric-phenotype cancer, whereas in intestinal-phenotype cancers, 5q allelic loss was more frequent. Mixed phenotype cancers have two distinct genetic types: either LOH or MSI types; MSI was only observed in mixed phenotype cancers. Overexpression of p53 gene is common in intestinal-type cancers, this is thought to play a fundamental role in the carcinogenesis of early adenocarcinomas of the stomach [33].

Cunningham *et al*. [3] investigated whether any genes up-regulated in gastric adenocarcinoma were also expressed in precursor lesions. Three target genes were studied; claudin-4, mitogen-activated protein kinase kinase 4 (mkk4) and 14-3-3σ (stratifin). Claudin-4 (cldn4) is a member of the claudin gene family that includes the genes claudin-1 (cldn1) and claudin-7 (cldn7) which have been shown to be up-regulated in a variety of tumors and their associated premalignant lesions, including colorectal cancer and cancers of the pancreas and oesophagus [3, 36]. Expression of cldn7 in a tff1 knockout (-/-) mouse model is weak during IM, but becomes strongly expressed and over-expressed in dysplasia and intestinal-type adenocarcinoma, respectively. No overexpression was detected in diffuse-type carcinoma suggesting that cldn7 would be useful marker for early intestinal-type GC [36]. Mkk4 is thought to be a tumor-suppressor gene; it is mutated in approximately 4% of many tumor types including pancreas, biliary, breast and colon. Stratifin is involved in diverse cellular processes including cell cycle progression, apoptosis, signal transduction and stress response. There are conflicting reports on its role in cancers as it has been reported to be up-regulated in pancreas and lung cancer; yet down-regulated in breast, prostate and liver cancer. The expression pattern of these proteins in normal gastric mucosa, premalignant intestinal metaplasia and gastric epithelial dysplasia, invasive gastric adenocarcinoma, and gastric adenocarcinoma metastases was studied. Immunohistochemistry assays were performed using tissue microarrays; all three proteins were positive markers of IM and adenocarcinoma. The reported findings present a panel of three markers which may provide nearly 100% sensitivity and specificity in identifying the gastric adenocarcinoma precursor lesions intestinal metaplasia and gastric epithelial dysplasia. Potential future application of this is suggested endoscopically, whereby labelled antibodies could be used to detect precursor lesions during the endoscopic procedure as a screening method for patients at increased risk for adenocarcinoma [3].

### 2.4 Progression of IM into Gastric Adenocarcinoma

Studies using cdx-2 transgenic mice have defined a key role of IM directly leading to malignant and invasive gastric adenocarcinoma [28]. Many epidemiological studies have found an association between the formation of IM and the development of GC, yet there was little direct evidence that IM is, in fact, a precursor lesion for gastric carcinoma. However, it was demonstrated that the gastric fundic mucosa of cdx-2 transgenic mice was completely changed into intestinal metaplastic mucosa [25,  28]. Further pathological examination of the intestinal metaplastic mucosa of the cdx-2 transgenic mice revealed formation of gastric polyps in 100% of the mice after 80 weeks versus no polyps in non-transgenic mice of the same age. Immunohistochemical staining was used to determine the type of gastric adenocarcinoma and clarify the activation of a molecular signalling pathway during carcinogenesis known as the Wnt signalling pathway. Indicators for activation of this pathway were detected by staining for nuclear translocation of β-catenin in the polyp epithelium and the presence of mutations in the apc and p53 genes. The transgenic mice that carried apc mutation or p53 deficiency developed gastric polyps at a much earlier age thus indicating the importance of mutations in these genes during gastric carcinogenesis. The authors concluded that IM is a precancerous lesion leading directly to GC in this model [28]. Cdx-2 expression in stomach cancer may be a marker of the progression of gastric carcinogenesis, and its activation may represent an early event [1]. Previous studies by Tsukamoto *et al. *[21,  24] also identified the ectopic expression of the cdx genes and the down-regulation of sox2 as important steps in gastric carcinogenesis progressing from IM which could represent important diagnostic and/or therapeutic targets.

It was demonstrated that the origin of GC cells in cdx2 transgenic mice is not parietal cells, but intestinal metaplastic epithelial cells that are entirely changed from gastric epithelial cells by cdx2 [28]. Although cdx1 induces gastric intestinal metaplasia, this differs from cdx-2 induced IM in differentiation, structure and proliferation resulting in the discovery of significantly thicker metaplastic mucosa in cdx1 transgenic mice than that in cdx2 transgenic mice [29]. It is widely believed that the phenotypic expression of tumor cells is the same as that of the tissue of origin of the cells [19]. However, this conflicts with Lauren’s histological classification of cancers as the gastric or “G” type of recent classifications correlates with the “intestinal” type of Lauren, and the “I” type in more recent classification correlates with the “diffuse” type of Lauren. The improved IM classification system is needed to allow for studies of the histogenesis of GC and phenotypic expression at the cellular level [19].

Expression levels of li-cadherin (cdh17) were reported to be significantly higher in chronic atrophic gastritis with IM than in GC, and lower in poorly differentiated tumors than in well- and moderately differentiated tumors, as well as showing increased expression levels in lymph node metastasis. No li-cadherin is expressed in normal tissues and expression levels were correlated with the GC differentiation grade. This suggests that overexpression of li-cadherin is an early event in gastric carcinogenesis and expression levels are associated with invasiveness and lymph node metastasis. Li-cadherin thus represents a useful biomarker for GC, as an early detection marker and also a discriminator of invasiveness, lymph node metastasis and potentially, patient prognosis [37].

## 3 GENE REGULATION DURING THE PROGRESSION OF IM AND GASTRIC CARCINOGENESIS

Studies of transcriptional regulatory control and transactivation involving sox2, cdx1 and cdx2 have shown that the expression patterns of sox2 and cdx1/2 appear inversely related and the down-regulation of sox2 may be an important mechanism in IM development. It is suggested that either sox2 may negatively regulate cdx1/2 expression or vice versa [21,  24].

More recent studies demonstrate that Cdx2 drives the transcription of furin during intestinal epithelial cell differentiation [31] and that the gene oct-1 is over-expressed in intestinal metaplasia, and binds to, but does not transactivate, cdx2 in gastric cells [38]. Furin is a calcium-dependent serine protease that processes many proteins including transforming growth factor-β1 (Tgf-β1), Bmp-4 and the cell adhesion protein E-cadherin. The fur gene, which encodes furin, is driven by three promoters P1, P1A and P1B and the cdx2 gene has been shown to induce the P1 promoter through a specific cdx2-DNA binding element. Furin expression is significantly enhanced during differentiation of intestinal epithelial cells and it is suggested that the inhibition of E-cadherin maturation by furin may affect intestinal epithelial cell differentiation [31]. Oct-1 is a ubiquitously expressed transcription factor that has been implicated in the activation of the mouse cdx2 promoter in pancreatic and intestinal cell lines. A significant association was observed between oct-1 and cdx2 expression in the gastric carcinoma cell lines, GP220 and MKN45; however, whilst oct-1 was shown to bind to the cdx2 promoter, a direct effect of oct-1 in the transactivation of cdx2 was not demonstrated [38].

## 4 GENETIC RISK FACTORS ASSOCIATED WITH IM/GC

*Helicobacter pylori* infection is a major risk factor for GC and induces many gene expression alterations by initiating an inflammatory response, so as a direct result many cytokines and other pro-inflammatory genes are upregulated. A number of polymorphisms have been identified in the pro-inflammatory cytokine genes which are related to an increased risk of developing GC. The main genes which have polymorphisms associated with greater risk of GC are the il-1 gene cluster, tnf-α and il-10 [39-43]. Many cytokine gene polymorphisms influence mucosal cytokine expression, gastric inflammation and the long term development of precancerous lesions in *H. pylori* infection. They have all been described and reviewed comprehensively and will not be discussed in further detail in the present review [39-45]. A Chinese study has reported that a polymorphism in cyp2e1/dra1 interacts with smoking to increase the risk of advanced precursor lesions on the pathway to GC [46].

## 5 MOLECULAR EVENTS AND ABERRANT GENE EXPRESSION IN GASTRIC CARCINOGENESIS

Studies of many types of cancer including GC have so far shown that many genetic and epigenetic changes take place during tumor progression [10,  47,  48]. As previously discussed, some initial genetic changes in intestinal-type GC can be detected in pre-malignant IM and dysplasia that are often involved in the transition of cells from a gastric to an intestinal phenotype, such as the cdx and muc genes. Many genes have previously been identified as deregulated in some way in GC resulting from a multitude of causes; this differs in the two types of GC, diffuse- and intestinal-type. Alterations occur at the level of the genome, at the level of signalling, and at the level of transcription and translation. The pathways of molecular events in the two types of GC have been reviewed comprehensively [10,  12,  17,  47], and Fig. (**3**) shows an overview of the main genetic changes in intestinal-type GC [12]. This review will focus on changes specifically important in intestinal-type GC and its precursor lesions and discuss genomic expression profiling studies which have highlighted important results for this type of cancer.

There are two general, classical groups of gene types involved in gastric carcinogenesis, the first are oncogenes. This group includes a wide range of genes that are overexpressed and commonly have a role in growth, differentiation and proliferation. Some of these genes are present at an early stage during carcinogenesis, and many are more prominent during tumor progression and are involved in metastasis and angiogenesis. The second group are known as the tumor-suppressor genes (TSGs), these genes are all silenced or deactivated by a variety of mechanisms and often have roles in cell cycle regulation and cell death (apoptosis). Tables **1** and **2** provide shortlists of the main oncogenes (Table **1**) and TSGs (Table **2**) as well as other genes that have aberrant expression and/or mutations involved in the progression of IM and intestinal-type GC that are discussed in this review. All the oncogenes and TSGs in the two types of GC have been reviewed comprehensively as stated above, the following section aims to highlight some of the more important changes in both types of GC incorporating many important aberrantly expressed genes that are detected at the precursor stages of intestinal-type GC. Recent expansion of the two classical groups of oncogenes and TSGs has yielded a third category of genes; the microRNA (miRNA) family of regulatory molecules [49]. More than 50% of miRNA genes are located in cancer-associated genomic regions known as fragile sites; evidence suggests that miRNAs function in concert with classical tumor suppressors and oncoproteins to regulate key pathways involved in cellular growth. When such pathways become deregulated, miRNAs and their targets play a key role in tumorigenesis [49,  50].

### 5.1 Oncogenes

Gene over-expression has been observed in many GC oncogenes including k-ras, c-erbB2, c-met and k-sam [12,  47]. Of these, c-met and k-sam are more commonly amplified in diffuse-type GC, whereas c-erbB2 over-expression and k-ras mutation are seen in intestinal-type GC but not diffuse-type. K-ras has also been reported in the precursor lesions IM and adenoma [12,  17,  51], yet has been shown to decrease after *H. pylori* eradication suggesting that *H. pylori* clearance therapy before the formation of stable k-ras mutations will decrease the risk of GC [52]. Further well-characterized oncogenes include c-myc at 8q24, ErBB2 at 17q12 and cyclin D at 11q13 [53,  54]. The c-myc gene is a regulator of cell cycle and plays a major role in control of cell growth, differentiation, apoptosis and neoplastic alteration, its overexpression is a common alteration in many cancers and has been reported in gastric neoplasias [55]. Distinct patterns of c-myc alterations have been shown between intestinal and diffuse-type cancers and c-myc locus amplification has been implicated as a predictor of aggressiveness in intestinal-type GC [55,  56]. Recent studies have demonstrated an oncogenic role of c-myc involved in regulation of the miR-17 microRNA cluster, which is further discussed in section 5.3 [49,  50,  57-62]. Cyclins condition the course of a cell cycle through the activation of appropriate serine-threonine kinases, cyclin D1 and E are found to be overexpressed in GC [63,  64]. Cyclin D1 is a key cell cycle regulator that may be useful in the diagnosis of early gastric carcinoma [65,  66]. Cyclin E overexpression has been associated with a high incidence of lymph node metastasis, and its expression was inversely correlated with a 5-year survival rate, suggesting that this gene is a useful prognostic indicator, but similar results were not obtained for cyclin D1 [63,  64]. Cyclin E overexpression also correlated with reduced expression of the TSGs p53 and p21 [63], but no correlation was found with expression levels of the TSG p27 in contrast to previous studies [12].

A systematic analysis of the 17q amplicon revealed the overexpression of 8 genes in GC [53]. The 17q amplicon contains the brca1 locus involved in breast cancer at chromosome 17q21; this chromosome also contains a potential TSG in GC [67]. Of note amongst the 8 overexpressed genes were estAA552509, top2A (both in 82% of tumors) and erb-B2 (30% of tumors). The role of top2A and erb-B2 in breast cancer is well-known; top2A is an enzyme that catalyzes functions in DNA replication and is a molecular target for many anti-cancer drugs (topo2 inhibitors); erb-B2 is amplified frequently in breast cancer and is an independent prognostic target. Co-amplification of top2A and erb-B2 is often reported in breast cancers, yet in gastric adenocarcinoma, overexpression of top2A was independent and more frequent than of erb-B2 [53]. A cluster of 5 tightly regulated genes were all present at chromosome 19q12, including ccne1. Other genes within the 19q12 amplicon were overexpressed including genes involved in cell cycle progression and cell proliferation, for example; cdc2, centromere protein A (cenpA), translin (tsn), tubulin beta 2 (tubb2) and bub1. Overall, high expression of the 19q12 gene cluster was statistically correlated with the cell proliferation gene signature, demonstrating an oncogenic role of this amplicon in GC [54].

The Egf family of growth factors and cytokines (includes Egf, Tgfα, IgfII and bFgf) are commonly over-expressed in intestinal-type GC. GC cells express neutrophilin-1 (nrp-1), a co-receptor for vascular endothelial growth factor (Vegf) receptor 2 endothelial cells; Egf induces both nrp-1 and Vegf [12]. Vegf has a prominent role in tumor angiogenesis, growth and metastasis [68,  69], and its expression correlates with disease recurrence in patients with early gastric carcinoma [70]. The vegf gene encodes a multifunctional cytokine that is crucial in angiogenesis during the development of cancer and activates p38 Kinase, which in turn, mediates actin reorganization and cell migration in human endothelial cells. p38 may thus also be an important regulator of angiogenesis [70]. Vegf-C is a member of the Vegf family that can induce lymphangiogenesis, a critical factor in the progression of many malignant tumors. Vegf-C and lymphatic vessel density (LVD) are related to lymph node metastasis (LNM) and poor prognosis of patients in GC, and serum vascular endothelial growth factor-C (SVegf-C) has been recently proposed as a useful biomarker for LNM and prognosis in GC [69]. Fgf7 is a mesenchyme-derived growth factor that is expressed in normal adult stomach, it acts specifically on cells of epithelial origin, stimulating their migration, differentiation and proliferation. Expression levels are markedly elevated in gastric inflammation and cancer [71].

The matrix metalloproteinases (Mmps) are key players in the promotion of cancer progression by increasing cancer-cell growth, migration, invasion, metastasis and angiogenesis [72,  73]. They function in the cell as primary matrix degrading proteases, collectively able to degrade all protein components of the extracellular matrix [73]. Microarray studies have revealed the increased expression of mmp1, mmp2, mmp3, mmp7, mmp9, mmp10, mmp11, mmp12, mmp13 and mmp14 in GC [8,  70,  74-76].

Cell-adhesion and metastasis-related genes are another subset of genes involved in carcinogenesis. E-cadherin is a commonly mutated cell adhesion gene, but is more often linked with diffuse-type cancer than intestinal-type. E-cadherin binds β-catenin in normal cell functioning; however, deregulation of the β-catenin pathway is associated with the loss of this binding, resulting in nuclear accumulation of β-catenin [77,  78]. The *H. pylori* cagA gene has been shown to be one of the mechanisms of this deregulation, by interacting with E-cadherin itself and causing consequent deregulation of the β-catenin pathway [78].

Other genes involved in gastric carcinogenesis, disease progression and survival are hypoxia-inducible factor-1α (Hif-1α) [11], the novel gene urg4 [79], r-ras [80] fhl2 [81], and the ribosomal protein L15, which has been associated with cell proliferation in GC, outlining its potential as a therapeutic target [82]. All GC cell lines and tissues exhibit abnormal cd44 transcripts containing the intron 9 sequence, a feature that has also been observed in 60% of gastric IM but is absent from normal mucosa [12].

### 5.2 Tumor Suppressor Genes (TSGs)

Cellular growth and proliferation are tightly regulated in cells; loss of regulation is a crucial early step in tumorigenesis. TSGs are often involved in apoptotic pathways which prevent cells being driven into an overactive proliferative state. Two pathways that are important in apoptosis are Wnt signal transduction and the β-catenin pathway. The Wnt signal transduction pathway plays an important role in gastric carcinogenesis and its activation has been reported in one-third of gastric adenocarcinomas. The activation of the Wnt pathway is frequently caused by β-catenin mutation which occurs in both intestinal- and diffuse- type GC [83]. In the cytoplasm, in the absence of Wnt, β-catenin is rapidly phosphorylated and degraded by Apc; however the stabilization of β-catenin in the cytoplasm due to apc mutation is a common event in GC. The β-catenin proteins accumulate in the cytoplasm and some translocate into the nucleus where they activate the Tcf/Lef (T cell factor/Lymphoid-enhancer factor) transcription factor. The β-catenin/Tcf complex subsequently activates a large number of oncogenes including vegf, c-myc, c-jun, fra-1, cyclin D1 and gastrin [84]. Nuclear localization and Wnt activation has been reported in approximately one-third of gastric adenocarcinomas, both intestinal and diffuse types [83]. The Wnt pathway is an important regulator of gastrointestinal stem cell proliferation and homeostasis; Wnt pathway activation is a key early event in multistep gastric carcinogenesis [83,  85]. Furthermore, a study on the localization and mutation of β-catenin in GC of different phenotypes demonstrated that nuclear accumulation and mutations in exon 3 of β-catenin correlate with intestinal phenotypic expression in gastric adenocarcinoma; no mutations were detected in cases with cytoplasmic or membranous localization. Early gastric adenocarcinoma mainly consists of gastric phenotype malignant cells with largely membranous localization of β-catenin, whereas advanced cancers have more malignant cells of the intestinal phenotype and nuclear β-catenin accumulates during the shift from the gastric to the intestinal phenotype [77]. Another major mechanism of Wnt signalling activation in GC is aberrant methylation and consequent silencing of the secreted frizzled-related protein (SFRP) family of genes; this may contribute to boosting cellular proliferation and inhibiting apoptosis although the mechanisms for this are unclear [86]. SFRP1 inactivation is a common, early event caused mainly by hypermethylation in GC; in one study, loss of expression was also correlated with tumor stage and lymph node status and was suggested to be associated with poor prognosis in GC patients [87].

The p53 TSG serves as a major cellular barrier against cancer development by way of apoptosis induction. P53 gene mutations and/or protein accumulation are the most common genetic changes in human tumors [88,  89]. The p53 gene is frequently inactivated by loss of heterozygosity (LOH), missense mutations and frameshift deletions, and in addition to the loss of wild-type p53, a high percentage of tumor cells accumulate mutant P53 protein isoforms [12,  88]. Whilst wild-type P53 functions as a tumor-suppressor, tumor-associated P53 proteins acquire novel functions enabling them to regulate gene expression and promote a large spectrum of cancer phenotypes [88]. P53 gene mutations are more frequent in intestinal than in diffuse type tumors, and alterations have been reported to occur at the premalignant stages of dysplasia and IM. Thus, p53 mutations have a crucial and early role in gastric carcinogenesis of the intestinal type; an immunohistochemical and genetic analysis revealed that early tumors with the poorest prognosis (i.e. that deeply penetrated the submucosa), showed the highest frequency of both p53 gene mutation and of nonmutated protein accumulation. However, p53 mutations and nuclear accumulation are late events in diffuse-type GC [89]. P53 belongs to a small family of sequence-specific nuclear transcription factors, to which p63 and p73 also belong [90]. P53 and P73 share structural and functional similarities; both are potent tumor suppressors with different isoforms that accumulate in response to DNA damage and have altered functions which often oppose those of the wild-type. Isoforms of P73 can inhibit both p53 and p73, thus assisting in tumor progression in contrast to the wild-type role of tumor suppression. In the absence of functional p53, p73 can be responsible for regulating the cell cycle, promoting apoptotic cell death in tumor cells and regulating the response of tumor cells to drugs; however, the regulatory mechanisms of the pro-apoptotic activity of p73 are distinct from those used by p53. The complexity of p73’s activity results in contradiction in its role as an important member of the p53 TSG family, thus further investigations are needed to fully verify its role in cancer [90,  91].

The Puma protein (p53-upregulated modulator of apoptosis) belongs to the BH3-only group of proteins along with Bcl-2. It is a potent mediator of the p53 apoptotic response that dimerizes with other BH3-only proteins resulting in release of cytochrome C from the mitochondria and induction of apoptosis by the activation of caspases 3 and 9 [84]. However, Puma expression is transactivated by p53 and p73 and its expression is increased in gastric carcinomas whilst remaining absent in normal gastric mucosa, suggesting it may have a potential oncogenic role and could possibly be used as a diagnostic marker [92].

RunX3 is a TSG which is frequently inactivated by allele loss or gene silencing due to promoter hypermethylation in GC and many other cancer types [93,  94]. RunX3 methylation has been detected in many premalignant lesions of gastric carcinomas, including 8.1% of chronic gastritis, 28.1% of intestinal metaplasia, 27.3% of gastric adenomas and 64% gastric carcinomas [94]. Deletion of the RUNX3 locus in mice results in hyperplasia of the gastric epithelium due to the stimulation of proliferation and suppression of apoptosis that is accompanied by a reduced sensitivity to TGF-β1. The tumor suppressor activity of RunX3 was demonstrated in nude mice when an increase in the level of runX3 expression resulted in decreased tumorigenicity. It has therefore been elucidated that RunX3 is a tumor suppressor of GC associated with the Tgf-β signalling pathway. Induction of apoptosis by Tgf-β is well-documented in many cancer cell types; however, the role RunX3 plays in Tgf-β-dependent cell cycle arrest and apoptosis is unclear [93]. A more recently proposed molecular mechanism for the anti-tumor activity of RunX3 is the inhibition of cancer angiogenesis, growth and metastasis. The growth and metastasis of GC depends on angiogenesis, the vascular endothelial growth factor, Vegf, has been identified as crucial to tumor angiogenesis. RunX3 was shown to directly suppress vegf gene transcription, which results in a significantly impaired angiogenic potential in human GC cells. RunX3 and Vegf expression were inversely associated and restoration of RunX3 expression in cancer cells inhibited tumor growth and metastasis [68].

Other TSGs commonly silenced by hypermethylation in GC are p14, p15, p16, p57, dap-kinase, hMLH1, e-cadherin and rassf1A [95-100]. The ink4a/arf locus encodes the two tumor suppressors, p16^INK4A ^and p14^ARF^, these are closely related genes located on chromosome region 9p21. P16^INK4A^ is a cell cycle regulator that inhibits G1-cyclin-dependent kinases, Cdk4 and Cdk6, and induces a G1-phase arrest. P15 also inhibits Cdk4 which mediates cell cycle control, particularly in the Tgf-β-signalling pathway. Dap-kinase (death-associated protein kinase) is a pro-apoptotic serine/threonine kinase involved in interferon-γ-induced apoptosis. hMLH1 is a DNA-mismatch repair gene and E-cadherin is a cell adhesion molecule that binds the  β-catenin pathway and has a role in the suppression of invasion and metastasis [97]. Hypermethylation of these TSGs has been detected in both GC and IM and a differential increase in methylation frequencies from premalignant to malignant tissues in dap-kinase, p14, p15 and p16 suggests accumulation of hypermethylation in these loci during progression from metaplasia to cancer [97]. Other reports of hypermethylation in GC suggest that it is an early event that often occurs in premalignant conditions such as IM and accumulates during tumor progression [48,  94,  101-103].

The cyclin-dependent kinase inhibitors p21 and p27 share 42% amino acid homology and whilst both had reduced expression in GC, the expression levels of each differed in different types of disease, with p27 loss associated with the sporadic form (Type III), and p21 loss associated with hypergastrinaemic cases (Type I or II) [104]. P21 functions as a cell growth cycle inhibitor and inhibits activities of the oncogenic cell cycle regulators, cyclins D1 and E [63]. A novel functional role of the tumor suppressor p21 has been proposed as an accelerant of Tgf-β1-mediated apoptosis in GC cells [105].

### 5.3 MicroRNAs

MicroRNAs are an important class of genes which function alongside TSGs and oncogenes and are involved in gene silencing and dysregulation during tumorigenesis. They are members of the small RNA family which include small interfering RNAs (siRNAS). Both micro RNAs (miRNAs) and small interfering RNAs (siRNAs) are evolutionary conserved, non-coding, small RNAs (21-25 nucleotides) involved in post-transcriptional gene regulation. About 200-300 miRNAs have currently been identified in humans and of these, about 50% are involved in cancer with either TSG or oncogenic functions [49,  50,  57-60,  106].

Some tumor suppressor miRNAs may be relevant in GC due to their interference with genes commonly deregulated in GC. The miRNAs miR-15a and miR-16a induce apoptosis by targeting Bcl2 [49,  60]; and the let-7 family of miRNAs have tumor suppressor activity by way of negative regulation of ras [49,  50,  57-59]. Let-7 and ras are reciprocally expressed in lung tumor tissue versus normal tissue where let-7 homologs were down-regulated and ras up-regulated; let-7 has been suggested as a potential therapeutic agent to treat lung cancer [58,  59].

The first of the oncogenic miRNAs that was shown to have tumorigenic activity was Bic/miR-155. This gene functions as an oncogene in cooperation with Myc and has been consistently observed in many tumor types including breast, lung, colon and thyroid cancers [49,  50], and may yet be relevant for GC. The microRNA miR-21 promotes tumorigenesis by inhibiting apoptosis and its overexpression has been reported in many tumor types including breast, colon, lung, pancreas, stomach and prostate [49,  50]. The miR-17 cluster refers to a miRNA polycistron located at chromosome 13q31, consists of a cluster of 6 human miRNAs (miR-17-5p, miR-18a, miR-19a, miR-20a, miR-19b-1 and miR-92), that has been implicated as an oncogene. It has been shown to be under direct control of c-myc, and the two function in concert to promote cell cycle progression and inhibit apoptosis [49,  50,  57-62]. Two miRNAs, miR-372 and miR-373, render cells insensitive to the effects of a normally functioning p53 allele by inhibiting expression of lats2 (large tumor suppressor homolog 2), this in turn relieves Cdk2 from repression, allows continuing cellular proliferation and overcomes cellular senescence [49,  50].

The P13K/Akt signalling pathway results in aggressive proliferation and is activated in most tumors, including gastric adenocarcinoma [107-109]. The tumor suppressor gene, pten (phosphatase and tensin homolog on chromosome ten), inhibits P13K function. Loss of pten expression is associated with P13/Akt signalling activation in many tumors. MiR-19a binds pten *in vitro*, and overexpression of miR-19 in lymphomas correlates with reduced expression of the Pten protein, thus overexpression of miR-19 in tumors could represent an alternative mechanism of activation of the P13K/Akt signalling pathway [58].

Loss or gain of function of specific miRNAs appears to be a key event in the genesis of diverse cancers including GC, such that miRNAs are now known to regulate pathways controlled by classic tumor suppressors and oncogenes including p53, myc and ras [49]; these genes are all involved in GC. [58,  59]. Thus, a tumor suppressor or oncogenic miRNA may represent an ideal target for diagnosis, prognosis and therapeutic intervention of many cancers.

Tumorigenesis involves the accumulation of multiple mutations and genetic changes and most human tumor cells contain multiple chromosomal abnormalities [110,  111]. Two major classes of genetic instability have been characterized: microsatellite instability and chromosomal instability [111].

### 5.4 Microsatellite Instability (MSI)

Microsatellite instability (MSI) results in defective DNA repair processes; it is a hallmark of the DNA mismatch repair deficiency that is one of the main pathways of gastric carcinogenesis. Microsatellites are short DNA sequence repeats found throughout the genome and nearly always present in GC cases associated with germline mutations of the mismatch repair (MMR) genes: hMsH2, hMLH1, hPMS1, hPMS2 and MSH6/GTBP [12]. In most gastric adenocarcinomas, MSI originates from inactivation of the hMLH1 gene promoter by hypermethylation [98,  112]. MSI of the D1S191 locus is found in 26% of IM and 46% of intestinal-type cancer [12]. Furthermore, a study on early gastric neoplasms demonstrated that hypermethylation-associated inactivation of the hMLH1 gene can occur in early gastric carcinogenesis and the MSI- and hypermethylation-associated inactivation of hMLH1 are more prevalent in early GC than in gastric adenoma [113].

### 5.5 Chromosomal Instability

Chromosomal instability involves losses of heterozygosity (LOH) and a likely cause of this is telomere dysfunction [111]. Telomeres are required for the stable transmission of linear chromosomes and in normal human somatic cells, and they shorten gradually with age. The telomere specific protein, Trf1, regulates telomere length, and Trf2 maintains telomere integrity [114]. The telomeres of germ line and cancer cells, however, do not shorten, this is maintained by telomerase by way of extending 3’ telomeric overhangs or recombination [115]. In GC, reduced levels of expression of a single-stranded telomeric DNA-binding protein, protection of telomeres (Pot1) has been associated with telomere dysfunction, and changes in Pot1 expression levels were potentially associated with stomach carcinogenesis and GC progression [115]. Most intestinal-type GCs have elevated telomerase activity and expression of hTERT (human telomerase reverse transcriptase; the enzyme that catalyzes telomere DNA synthesis) resulting in shortened telomere length; over 50% of IM expresses low levels of telomerase activity, equivalent to 10% that in GC [12].

### 5.6 DNA Methylation

One of the most frequent mechanisms of gene inactivation in GC is epigenetic silencing by DNA methylation. DNA methylation is a chemical modification that alters the phenotype of a gene without altering its DNA sequence. Gastric carcinoma is one of the tumors that has a high frequency of CpG island hypermethylation; CpG islands are short CpG-rich regions present within the promoter region of approximately 40-50% of human genes [101-103,  116]. The concordant methylation of multiple genes is termed as the CpG island methylation phenotype (CIMP); this is the phenotype most often associated with gastric carcinoma and is characterized by tumors methylated at multiple loci “methylated in tumors” known as the MINT loci (MINT1, MINT2, MINT12, MINT25 and MINT 31) [48, 103]. Hypermethylated CpG islands in gene promoters causes subsequent loss of transcription of the genes, referred to as gene silencing [103,  117,  118]. DNA methyltransferase 1 (Dnmt1) is the main enzyme involved in this [47,  118]. Tumor-suppressor and tumor-related genes known to be silenced in GC due to promoter hypermethylation include p14, p15, p16, p53, p57, hMLH1, cdh1 (e-cadherin), apc, chfr, cox2, dap-kinase, gstp1, hpp1, mgmt, rassf1A, runX3, timp-3 and thbs1 [10,  47,  48,  94,  98,  100-103,  116]. There are multiple reports of the association between MSI and hypermethylation; inactivation of the mismatch repair gene hMLH1 is known to cause MSI [98,  113,  117,  119]. Demethylation of CpG sites within genes, also known as hypomethylation, is also involved in GC progression *via *a process of activation of oncogenes including r-ras, elk1, frat2, fgfR2, rhoB and rho6 [80]. A study using siRNA in R-ras-expressing GC cell lines demonstrated that blocking the r-ras signalling pathway is a potentially successful candidate for GC therapy [80].

Hypermethylation in GC is reported to be an early event that accumulates during tumor progression [48,  94,  101-103]. Hypermethylation and consequent silencing of p16, p57 and runX3 have been shown to occur at the early stages of gastric carcinoma [93,  94,  100]. It has been reported that methylation of p16 may play a role in the malignant transformation of gastric precursor lesions; p16 methylation was present in 7% IM, in 18% adenomas unassociated with carcinoma, in 29% of adenomas/dysplasias associated with adenocarcinoma and, finally, in 44% adenocarcinomas [103]. Other genes predominantly methylated at the early carcinogenic stages of gastric carcinogenesis, including IM and dysplasia, are: dap-kinase, e-cadherin, p14, thbs1 and timp-3 [102].

Aberrant DNA methylation is more frequently present in GC than mutations, and as such presents a useful tool to identify novel TSGs [120]. *Helicobacter pylori* has been shown to induce aberrant methylation in gastric mucosae [121], and promoter hypermethylation is frequently detected in premalignant gastric IM, regardless of the presence or absence of tumor [97]. Aberrant methylation patterns represent useful biomarkers for cancer detection, the assessment of prognosis, as therapeutic targets and for the prediction of response to anti-tumor treatment; furthermore, they may be useful to detect past exposure to carcinogens such as *H. pylori* and assess future risk of cancer development [100,  120, 121].

## 6 GENE EXPRESSION STUDIES OF IM AND GC

At present, there are limited reports of human gene expression in IM, particularly using genome-wide and other molecular transcriptional-based studies, such as microarray technology and RT-PCR. As described in section 2, many expression studies have defined the morphological characteristics and identified the expression of certain genes histologically which are related to various types and stages of IM. Reports of expression profiles of other precancerous conditions have also been undertaken including studies of gene expression profiles associated with gastric hyperplastic polyps [122] and gastric adenoma [123]. Gene expression studies of gastric IM using cDNA microarrays include a study on inflammation, metaplasia and tumor development in mice [124], a study on IM versus GC tissues obtained from GC patients during resection [125] and studies of gene expression profiling of malignant and non-malignant lesions of the esophagus and stomach using fresh patient tissues [126,  127]. There are, however, numerous studies that present results of microarray gene expression profiling of GC, some comparing GC to normal tissues, some distinguishing the gene expression profile of the different types of cancer i.e. intestinal versus diffuse and a small number which compared GC to its precursor lesions. An overview of the most relevant microarray expression studies involving IM comparisons and clustering of different cancer types is included here.

### 6.1 Gene Expression Profiling of GC versus IM and other Precursor Lesions

The first report of microarray gene expression profiling of GC and its precursor lesions was a study of 91 Australian and 33 Chinese patients to distinguish the gene expression patterns related to clinical, pathological and ethnic parameters [125]. This study and many others have used microarray expression profiling to classify tissue samples into malignant, pre-malignant and non-malignant groups as well as distinguishing the two types of cancer (intestinal and diffuse) by expression profile. Data such as this may prove to be of significant use in diagnosis, prognosis and choice of treatment for future patients. Tumor and normal samples were collected from patients with GC requiring resection consisting of 65 tumor samples, 22 IM samples, 27 chronic gastritis samples and 9 normal tissue samples. Unsupervised clustering of the data distinguished malignant and non-malignant groups, and also different histological groups including chronic gastritis, IM and the different types of GC: intestinal, diffuse and mixed. A clear segregation of the GC samples was not evident between Australian and Chinese patients indicating a lack of ethnic differentiation in the molecular profile of GC. The chronic gastritis group was defined by a mitochondrial gene expression signature, in particular, nuclear genes encoding mitochondrial proteins including cytochrome c oxidase (Cox) and NADH dehydrogenase (Nduf). This may be linked to *H. pylori* infection, which is invariably present in this group. The expression profile of IM reflected expression of genes characteristic of the intestine representing a transition toward a transformed phenotype; these included cdx1, myo1A, the microsomal triglyceride transfer protein MTP, cholecystokinin, villin-1, fat and trefoil factor1, tff1. Many markers of mature intestinal cell function showed decreased expression or loss of expression in intestinal-type GC, possibly representing less differentiation in tumor tissues than in metaplastic tissues, this was evident in Tff1 which had reduced expression in over half of the intestinal and diffuse GC samples [125].

Other premalignant tissue types that have been studied using expression microarrays include Barrett’s Esophagus (BE), gastric polyps and adenoma. A microarray expression profiling study of resected human IM samples was used to investigate the classification of Barrett’s disease and associated conditions including IM, GC and adenocarcinoma of the esophagus and gastroesophageal junction (GEJ) [127]. Barrett’s esophagus describes the condition of IM in the esophagus which has a strong association with gastric IM; both are considered as lesions with higher risk of malignant transformation to adenocarcinoma in the esophagus and stomach, respectively, and both are associated with inflammatory stimuli. Specific functional modules were identified that had significant levels of altered gene expression: the glycolipid metabolism module and the cytokine-cytokine receptor interaction module. Genes within the cytokine-cytokine receptor module were expressed at high levels in samples of IM and Barrett’s oesophagus. A stronger correlation was observed between adenocarcinomas of the esophagus and IM of the stomach than with Barrett’s mucosa and all but one carcinoma of the GEJ clustered together with adenocarcinomas of the stomach. This led the authors to speculate that adenocarcinomas of the esophagus could originate at the cardia invading the esophagus from below. In summary, the study was able to select a small set of genes that define new hypotheses for the malignant transformation of IM to adenocarcinomas [127]. A previous study differentiated Barrett’s Esophagus (BE) compared to cardia intestinal metaplasia (CIM) using their expression profiles and suggested this might provide a new direction for diagnosis, therapy and prevention of BE [126]. Many other studies have been published of the gene expression profile and molecular events involved in Barrett’s Esophagus, [128-132] but as this is considered to be a precursor lesion for a separate type of adenocarcinoma than gastric IM, it will not be discussed in any further detail for the purpose of this review.

Microarray gene expression profiling was employed to study the chromosomal instability and consequent differential expression of 11 gastric polyps with intraepithelial neoplasia (dysplasia); three hyperplastic polyps and eight adenoma. Although only a minority of gastric cancers are likely to actually arise from adenomas, they provide a defined opportunity to survey chromosomal changes during the pathogenesis of GC. Hyperplastic polyps are thought to be reactive in nature rather than neoplastic, yet are occasionally implicated in the onset of GC. Gene expression profiles revealed that both types of polyp showed many chromosomal aberrations, several consistent with gastric malignant lesions, the most frequent was loss of 9p21.3. The authors suggest a link between the distinct aberrations for each type of polyp with two morphologically and genetically distinct pathways to GC; the hyperplastic polyp pathway and the (intestinal type) adenoma pathway [122]. A subsequent study to compare expression profiles of gastric adenoma with gastric adenocarcinoma identified three distinct gene clusters consistent with the grade of adenoma. Group 1 contained mainly small, low-grade adenomas characterized by gene expression consistent with normal stomach, group 2 contained mainly large low and high grade adenomas characterized by the overexpression of intestine-specific genes including the cdx2-regulated cdh17 and defa5. The upregulation of the intestinal-type cell lineage is implicated in the pathogenesis of intestinal-type adenocarcinoma. Group 3 contained mainly gastric adenocarcinoma characterized by overexpression of arg2, a gene that has been reported to be overexpressed in many cancers including gastric adenocarcinoma. The genes reported to be expressed in this study are different to the spectra that are usually reported in DNA microarray studies, it is suggested this may be due to the fact that many studies report on the gene expression of advanced stage carcinoma which may exhibit a different gene expression profile to the tissue studies here [123].

### 6.2 Expression Profiles of Diffuse-type Gastric Cancer (DGC) versus Intestinal-Type Gastric Cancer (IGC)

The Boussioutas study previously discussed [125] highlighted differential expression of genes involved in diffuse-type gastric cancer (DGC) versus intestinal-type gastric cancer (IGC). The gene expression signature of DGC reflected active extracellular matrix production and complex signalling and remodelling by cell growth regulators. DGC is characterized by a marked stromal reaction and genes encoding components of the extracellular matrix were overexpressed, including collagens, matrix metalloproteinases, osteoglycin and cadherin 11. Smooth muscle and cell adhesion molecule genes were upregulated as well as a number of genes encoding extracellular proteins that are linked to the regulation of cell proliferation, including secreted frizzled-related protein 4, Nmb, Dab2, SparC, Wnt5a, Dkk3 and Fdz1; however, many of these were also upregulated in IGC. Significant attenuation of Cdh1 expression was found in DGC compared to other tissues, this is a recognized feature of DGC and germline mutations of Cdh1 are associated with familial DGC [125,  133-135]. In contrast, the IGC expression profile was characterized by markers of cell division and proliferation, many of which were not elevated in DGC samples, including genes required for G2-M transition, DNA replication, spindle assembly and chromosome segregation. Many genes with differential expression have been associated with GC and other cancers in other reports, such as topoisomerase (top2A) and cdc25B [70,  74]. Classification of different types of cancer was performed in a subsequent study which describes distinct groups of molecular classifiers for different types of GC but few genes are named specifically [8]. Of note, diminished expression of the vhl gene in intestinal type adenocarcinomas and overexpression of matrix metalloproteinase (mmp2) were identified in the diffuse-type cancers. However, following hybridization to tissue arrays, Mmp2 was found to be overexpressed in both intestinal and diffuse-type cancers [8]. These data are supported by further studies which used clustering of gene expression profiles to distinguish DGC from IGC [136,  137]. Consistent with other studies, genes upregulated in diffuse versus intestinal type cancer included genes associated with cell adhesion or migration and the extracellular matrix, including sparC, tubB2, itgB1, arhgd1A, col1A1 and col1A2. The genes which were upregulated in intestinal type versus diffuse type GC included growth-factor receptor genes (grb10, isr2 and eps15R) and genes related to cellular proliferation and mitochondrial function (egfr, egr1, pcna, cdk2, psort, timm10 and bcat2). The different nature and mechanisms of the two types of cancer are represented by this data which, after further analysis, revealed 46 genes that clearly separated the two classes of tumor [137]. Hippo *et al. *[74] reported genes exclusively overexpressed in intestinal-type cancer following microarray expression analysis. Overexpression was reported of the intestinal enzyme genes reflective of intestinal differentiation (galc, guyc2 and gpx2), the intestinal differentiation marker li-cadherin (cdh17) of the cadherin family genes which have crucial roles in cell-cell adhesion, and c-erbB2 was exclusively expressed in intestinal-type cancer, thus reflecting gene amplification [74].

### 6.3 Expression Profiles of Malignant versus Non-Malignant Tissues

Molecular classifiers for GC and non-malignant diseases of the gastric mucosa, including IM, were identified using a cDNA microarray approach [8]. The study comprised of microarray expression profiling of 28 GC tissues (including 18 intestinal-type and 10 diffuse-type) and 71 non-tumor gastric tissue samples (including 28 normal, 21 gastritis and 22 IM) obtained during surgery or by endoscopy. Molecular classifiers were identified based on trios of genes defined by Fisher’s linear discriminant analysis; tissue samples were discriminated at the distinctive stages during the carcinogenesis cascade, the two types of adenocarcinoma, gastric and diffuse, were also discriminated. More genes were differentially expressed between tumor and normal samples than between IM and normal samples. No genes were found that could distinguish normal from gastritis tissue samples. Genes that had their expression progressively altered during the cascade of intestinal-type carcinogenesis (i.e. Correa’s cascade including metaplasia stage) were as follows: genes with increased expression; col1A1, fn1, ctsb, col1A2, hs.177781, daf and vim; genes with decreased expression were prpf8, hs.327751, vhl, lck, bad, vegfB and polr2H [8].

A microarray study of 22 GCs and 8 noncancerous gastric tissues identified 162 genes upregulated in cancer versus non-cancerous tissue which included genes known to be involved in GC. Immunohistochemical detection of high levels of β-catenin and e-cadherin supports other studies suggesting that mutation and nuclear accumulation of β-catenin is predominant in intestinal-type cancer and loss of e-cadherin is predominant in diffuse-type cancer [74]. Yu *et al. *[138] performed a microarray study of differential gene expression in GC, pericancerous epithelium and normal gastric mucosa. The study identified 35 genes upregulated in pericancerous epithelium and GC versus normal tissues, including cam-5 (CEA: cell-associated adhesion molecule 5) and cam-6. In pericancerous epithelium versus GC tissue, 6 genes were upregulated and 3 genes were downregulated [138]. CEA is a broad spectrum tumor marker which is suggested here to be a useful marker for GC and is reported elsewhere to be upregulated in GC [139]. Topoisomerase2 (top2) was also overexpressed versus normal tissue, this gene is involved in the increase in cell proliferation from S phase to G2/M phase of the cell cycle that has been reportedly overexpressed in previous GC studies [53,  70]. Significant upregulation of e-cadherin and integrin-β4 was demonstrated as consistent with previous reports [70, 74, 139], cell skeleton and external matrix protein genes were also upregulated consistent with previous studies [70, 74, 137]. These genes play an important role in tumor invasion [138].

### 6.4 Expression Profiling of Mouse Models of IM and GC

Gene expression studies have been used to assess the efficacy of mouse models of human gastric inflammation, metaplasia and tumor development. The gastrin-deficient mouse has been demonstrated to represent a new, useful model for how Helicobacter-negative achlorhydria predisposes to GC. Specific changes in gastric gene expression included activation of immune defense genes, interferon-regulated response genes and IM of the gastric mucosa was characterized by expression of intestinal-type genes including cdx1, cdx2, muc2, villin-1 and claudin 7 [124]. A profile of gene expression changes in Helicobacter-induced GC was demonstrated by infecting hypergastrinaemic mice with *H. felis* [76]. Gene expression alterations were characteristic of those seen in human GC samples including two growth factors that are known to be highly expressed in human GCs, reg1 and amphiregulin [140, 141]; members of the matrix metalloproteinase family, mmp-10 and mmp-13; and claudin-7 [19, 21, 24]. Genes consistent with *H. pylori* infection and hypergastrinaemia exhibited altered expression, and cytokines involved in the inflammatory response had altered expression, including upregulation of expression of the Th1 cytokines interferon-γ, il-1β and tnf-α. Deregulation of these genes in the mouse model mimics the pathway towards GC in humans following *H. pylori* infection [76].

### 6.5 Microarray Expression Profiling Summary

Microarray expression profiling has been used extensively in studies of GC, with results between investigators showing consistency in the majority of cases. Not all reports agree on all the same genes but there is greater consistency in finding general groups and families of genes with aberrant expression in certain tissue types, for example, the upregulation of genes involved in proliferation in intestinal-type GC. It could be argued that most cancer types would yield a higher expression of proliferation marker genes; however, many studies have been successful in using microarray technology to distinguish the different types of cancer. A consensus has been reached between numerous investigators regarding this, and a general expression profile of diffuse-type cancer that is very different to the intestinal type is largely agreed. For this reason, many microarray studies have suggested their use for predicting the diagnosis [142], prognosis and metastatic potential of GC [137, 143], and also to possibly determine survival and choice of appropriate therapy [75]. Furthermore, a study that used microarray expression profiling to investigate gene expression in gastric adenomas proposed that their results may be capable of defining subgroups of adenoma that are unresolved by conventional histopathology which may have a prognostic value should a follow-up study be undertaken [123].

Whilst microarray expression profiling of GC has been demonstrated to have a very low level of variability within the same tumor sample [144], this variability may be more pronounced when using other techniques and methodologies. A group in Japan used SAGE analysis with confirmation by RT-PCR to examine the expression profiles of GC and lymph node metastatic tissue. They identified changes in some genes previously reported in GC, as well as some novel gene expression alterations. They suggest that gene expression patterns of GC in Japan may be different from those in USA and Europe [139]. This is an interesting viewpoint that conflicts with previous research that investigated a geographic variation in GC expression profiles and found no difference [125]. This is the only report that suggests differences in data may reflect geographical differences [139]. Differences in methodology and techniques between laboratories is a likely factor contributing to these differences; an alternative explanation may be due to the differences that exist between Japanese and Western gastrointestinal pathologists in classification of dysplasia and cancer [145]. In summary, microarrays are proving to be very useful for classifying different tumor and tissue types, they may yet be useful for prognosis, diagnosis and treatment choices, but investigators must proceed with caution due to the problems in interpretation and consistency of microarray data as highlighted above.

## 7 SUMMARY AND FUTURE DIRECTIONS

This review has attempted to highlight the current situation of the known molecular events involved in the initial development of intestinal metaplasia and its subsequent progression into gastric cancer. Whole-genome microarray expression profiling has proven to be a very useful methodology for defining subgroups within GC and defining malignant tissue from non-malignant tissue; however, this technique has not as yet managed to expand on the intestinal-cell type markers of IM that have been very successfully defined by immunohistochemical analyses. Most markers appear to differentiate either intestinal-type or gastric-type cells and the expression of these progressively increases or decreases accordingly throughout the development of IM and GC, including such genes as the intestinal markers cdx1, cdx2 and cdh17, and the gastric marker gene, sox2. Thus, genes which have high levels of expression in IM and GC that could be potential markers of IM malignancy, have not been reported from immunohistochemical or whole-genome microarray analysis. The most useful technique to date, which has been able to identify genes with the same aberrant expression patterns in IM and GC, is methylation profiling. Many hypermethylated genes are known to be highly expressed in IM and GC, these include p53, p16 and runX3. DNA methylation is thus a key molecular mechanism in the development of GC and its presence links precursor lesions such as IM with the development of tumor tissues. The presence of miRNAs is also a new, exciting field which could yield enormous insights into the molecular mechanisms of aberrantly expressed genes in IM and GC. Research into this field and its role in cancer is still in the early stages, yet already, genes associated with IM and GC have been implicated as having microRNA interference in their regulation, including p53 and c-myc.

Future applications of new and improved technologies may yet be able to provide useful genetic markers which could allow for early detection of GC, by enabling diagnosis at the precursor stage of IM. Indeed, one of the immunohistochemical studies using suggested potential future endoscopic application of the IM and dysplasia detection markers Claudin-4, Mkk4 and Stratifin, whereby labelled antibodies could be used to identify precursor lesions during the endoscopic procedure as a rapid screening method [3]. Further research is needed into potential molecular markers of GC at the precursor lesion stage and the application of marker detection techniques in a clinical environment. There is considerable potential to exploit miRNAs and gene hyper- and hypo-methylation for diagnosis and therapeutic intervention of cancer. A potential therapeutic intervention that has been proposed to date is by targeting miRNA regulation with antisense oligonucleotides, referred to as anti-miRNA nucleotides (AMOs), that are complementary to mature oncogenic miRNAs and might effectively inactivate miRNAs in tumors and slow tumor growth [58, 59]. Future progress in the above research fields, incorporating both studies at the whole-genome level and studies of specific genes and their regulatory pathways, are hoped to further define and clarify the precise pathways of molecular and genetic events involved in the development of IM and subsequent progression into GC. The translation of these findings into a clinical setting for diagnosis and treatment of GC will maximize the future potential of this research.

## Figures and Tables

**Fig. (1):**
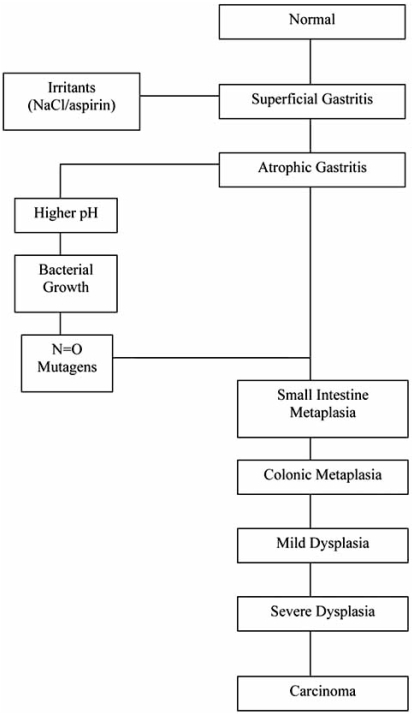
The Correa’s Cascade of Carcinogenesis [16].

**Fig. (2):**
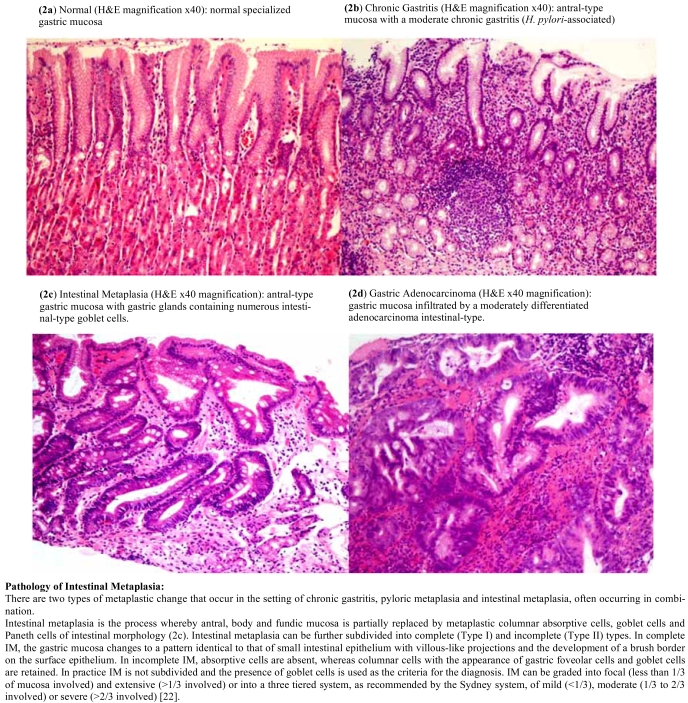
H&E Stained Histopathology Sections of a) Normal Gastric Mucosa, b) Gastritis, c) Intestinal Metaplasia and d) Intestinal-Type Gastric Cancer.

**Fig. (3):**
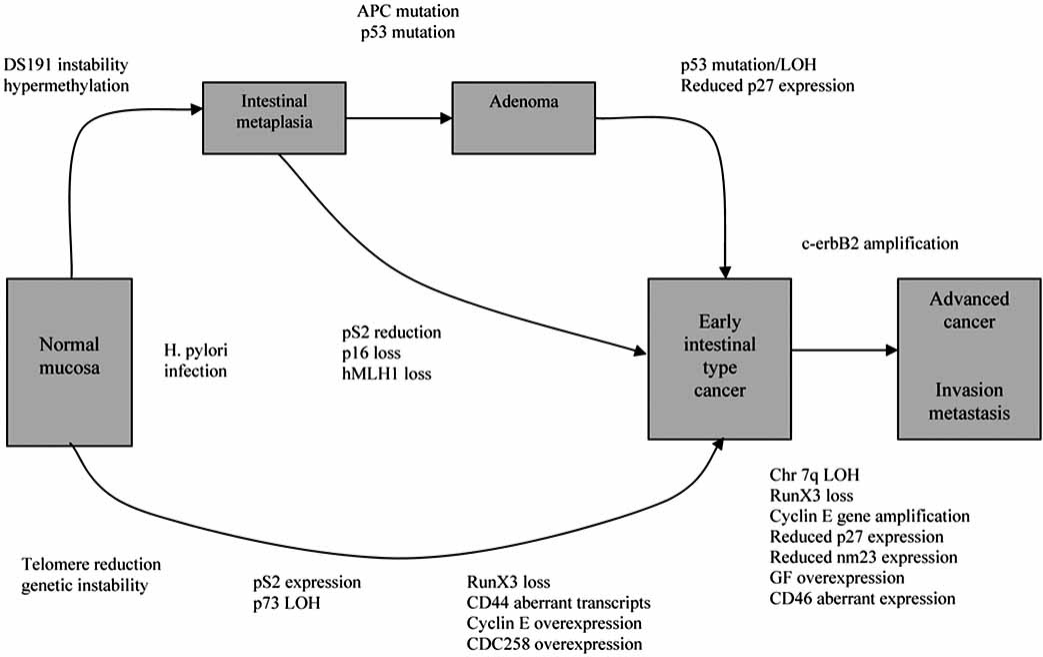
Schematic representation of the main genetic and epigenetic changes involved in the development of intestinal-type gastric cancer, reproduced with some modifications from Smith *et al*. 2006 [12].

**Table 1.** Oncogenes and Other Genes Aberrantly Overexpressed in IM and Intestinal-Type GC and their Functional Role

Gene Name	Expression During Intestinal Differentiation	Expression in IM	Expression in GC	Functional Role in IM/GC
Cdx1	+ +	+ + +	+	Regulator of intestinal cell lineage differentiation
Cdx2	+ +	+ + +	+	Regulator of intestinal cell lineage differentiation
Muc2	+ +	+ + +	+	Associated with intestinal cell lineage differentiation
Cldn4 (claudin-4)	+	+ + +	+ + +	Associated with intestinal cell lineage differentiation
Cldn7 (claudin-7)	+	+	+ + +	Associated with intestinal cell lineage differentiation
Mkk-4	+	+ + +	+ + +	TSG associated with intestinal cell lineage differentiation
Stratifin	+	+ + +	+ + +	Associated with intestinal cell lineage differentiation
Villin-1	+	+ + +	+ + +	Associated with intestinal cell lineage differentiation
Hif-1α	+ +	+ + +	+	Associated with intestinal cell lineage differentiation
Fabpi	+ +	+ + +	+	Associated with intestinal cell lineage differentiation
Tff1 (trefoil factor 1)	+ +	+ + +	+	Associated with intestinal cell lineage differentiation
Tff3 (trefoil factor 3)	+ +	+ + +	+	Associated with intestinal cell lineage differentiation
Gcc	+ +	+ + +	+	Associated with intestinal cell lineage differentiation
Cdh17 (li-cadherin)	+ +	+ + +	+ +	Cdx-2 regulated intestine-specific gene, associated with invasiveness and LNM
Defa5		+ + +	+ +	Cdx2-regulated intestine-specific gene
Furin	+ +	+ + +	+ +	Essential for morphological differentiation of intestinal cells
Oct-1	+ +	+ + +	+	Transcription factor, binds cdx2 promoter
Myo1A	+ +	+ + +	+	Intestinal-transformation specific indicator gene
Mtp	+ +	+ + +	+	Intestinal-transformation specific indicator gene
Fat	+ +	+ + +	+	Intestinal-transformation specific indicator gene
P53 (mutants)	+	+ +	+ + +	Regulate gene expression and promoted carcinogenesis
P73 (mutants)	+	+ +	+ + +	Inhibit WT p53 and p73 and promote tumor progression
K-ras (mutation)		+ +	+ + +	Oncogene, frequently mutated in intestinal-type GC
C-myc		+ +	+ + +	Cell cycle regulator, associated with tumor aggressiveness
C-erbB2			+ + +	Overexpression involved in gene amplification, poor prognosis and metastases
Cyclin D1			+ + +	Cell cycle regulator
Cyclin E			+ + +	Cell cycle regulator, associated with lymph node metastases
Top2A			+ + +	Involved in cell cycle and proliferation
Cdc2			+ + +	Promotes cell cycle and proliferation
Mmp (1-3, 7, 9-14)			+ + +	Promote angiogenesis and proliferation in GC
CD44		+ +	+ + +	Transmembrane glycoprotein; involved in cell-cell matrix interaction
β-catenin		+	+ + +	Accumulates due to APC mutation, activates many oncogenes
Vegf			+ + +	Growth factor, mediator of tumor angiogenesis
Pot1			+ + +	Associated with telomere dysfunction and LOH
hTERT		+ +	+ + +	Associated with telomere dysfunction and LOH
miR-17 cluster			+ + +	Drives cell cycle progression and inhibits apoptosis; acts in concert with c-myc
miR-372			+ + +	Render cells insensitive to wild type p53
miR-373			+ + +	Render cells insensitive to wild type p53

**Key to Table 1:** +/+ +/+ + + refers to gradual increase in expression/mutation

+ Expressed

++ Upregulated

+++ Overexpressed

**Table 2.** TSGs and Other Genes Aberrantly Expressed and Silenced in IM and Intestinal-Type GC and their Functional Role

Gene Name	Expression During Intestinal Differentiation	Expression in IM	Expression in GC	Functional Role in IM/GC
Muc1	-	- -	- - -	Associated with gastric-type cell lineage
Muc5AC	-	- -	- - -	Associated with gastric-type cell lineage
Muc6	-	- -	- - -	Associated with gastric-type cell lineage
Sox2	-	- -	- - -	Associated with gastric-type cell lineage
P53 (wild-type)	-	- -	- - -	Induces apoptosis, inactivated by LOH
P73 (wild type)	-	- -	- - -	TSG, induces apoptosis, belongs to p53 family
RunX3	-	- -	- - -	TSG silenced by hypermethylation; initiates Tgf-β-induced apoptosis, suppresses Vegf
P21 (WAF1/CIP1)			- - -	Cell growth cycle inhibitor, inhibits cyclins D1 and E
P27 (KIP1)			- - -	Cell growth cycle inhibitor, inhibits cyclins D1 and E
Apc (mutation)		-	- - -	TSG: degrades β-catenin
Tgf-β-signalling pathway			- - -	TSG: Induces apoptosis
P14 (ARF)		- -	- - -	TSG: regulates cell cycle, silenced by hypermethylation
P15 (INK4B)		- -	- - -	TSG: regulates cell cycle, silenced by hypermethylation
P16 (INK4A)		- -	- - -	Cell cycle regulator, induces G1-phase arrest, silenced by hypermethylation
P57 (KIP2)		- -	- - -	Inhibits cyclin-dependent kinases, silenced by hypermethylation
Dap-kinase		- -	- - -	Death-associated protein kinase involved in apoptosis, silenced by hypermethylation
hMLH1		-	- - -	DNA mismatch repair gene inactivated by hypermethylation; causes MSI

**Key to Table 2:** -/- -/- - - refers to gradual decrease in expression/mutation

- Minimal/reduced expression

- - Downregulated

- - - Silenced

## ABBREVIATIONS

IM: = Intestinal Metaplasia
GC: = Gastric Cancer
IL-1: = Interleukin-1
TNF-α: = Tumor Necrosis Factor-α
TSG: = Tumor Suppressor Gene
DGC: = Diffuse-Type Gastric Cancer
IGC: = Intestinal-Type gastric Cancer
